# Human iPSC Models to Study Orphan Diseases: Muscular Dystrophies

**DOI:** 10.1007/s40778-018-0145-5

**Published:** 2018-10-04

**Authors:** Guangbin Xia, Naohiro Terada, Tetsuo Ashizawa

**Affiliations:** 1 Department of Neurology, College of Medicine, University of New Mexico, Albuquerque, NM USA; 2 Department of Pathology, Immunology & Laboratory Medicine, College of Medicine, Gainesville, FL USA; 3 Houston Methodist Neurological Institute and Research Institute, 6670 Bertner Ave R11-117, Houston, TX USA

**Keywords:** Induced pluripotent stem cells, Muscular dystrophy, Model, Cell-based therapy, Genome editing

## Abstract

**Purpose of Review:**

Muscular dystrophies (MDs) are a spectrum of muscle disorders, which are caused by a number of gene mutations. The studies of MDs are limited due to lack of appropriate models, except for Duchenne muscular dystrophy (DMD), myotonic dystrophy type 1 (DM1), facioscapulohumeral muscular dystrophy (FSHD), and certain type of limb-girdle muscular dystrophy (LGMD). Human induced pluripotent stem cell (iPSC) technologies are emerging to offer a useful model for mechanistic studies, drug discovery, and cell-based therapy to supplement in vivo animal models. This review will focus on current applications of iPSC as disease models of MDs for studies of pathogenic mechanisms and therapeutic development.

**Recent Findings:**

Many and more human disease-specific iPSCs have been or being established, which carry the natural mutation of MDs with human genomic background. These iPSCs can be differentiated into specific cell types affected in a particular MDs such as skeletal muscle progenitor cells, skeletal muscle fibers, and cardiomyocytes. Human iPSCs are particularly useful for studies of the pathogenicity at the early stage or developmental phase of MDs. High-throughput screening using disease-specific human iPSCs has become a powerful technology in drug discovery. While MD iPSCs have been generated for cell-based replacement therapy, recent advances in genome editing technologies enabled correction of genetic mutations in these cells in culture, raising hope for in vivo genome therapy, which offers a fundamental cure for these daunting inherited MDs.

**Summary:**

Human disease-specific iPSC models for MDs are emerging as an additional tool to current disease models for elucidating disease mechanisms and developing therapeutic intervention.

## Introduction of Muscular Dystrophies

Muscular dystrophies (MD) are a spectrum of inherited, progressive muscle diseases. The terminal pathology often shows necrosis of muscles and replacement by fibrotic or fatty tissues. There are autosomal dominant, autosomal recessive, and X-linked muscular dystrophy. Dominantly inherited MDs are largely caused by gain-of-function mechanisms, while recessive MDs are primarily caused by loss of function. The proteins that are involved in MDs are localizable to extracellular matrix, sarcolemma, sarcomere, and myonuclei as well as nonstructural enzymes. The current trend is to classify MDs by the responsible genes, for example, sarcoglycanopathies, dystrophinopathies, dysferlinopathies, caveolinopathies, desminopathies, calpainopathies, and dystroglycanopathy. The most common MD is Duchenne muscular dystrophy/Becker muscular dystrophy (DMD/BMD) with a prevalence of 1.52 per 10,000 boys ages 5–9 from 2006 to 2010 [1]. Myotonic dystrophy type 1 (DM1) is the most common adult-onset MD with a prevalence of 10/100,000 [2–4]. Within limb-girdle muscular dystrophy (LGMD), the relevant prevalence is 12% calpainopathy, 18% dysferlinopathy, 15% sarcoglycanopathy, 15% dystroglycanopathy, and 1.5% caveolinopathy [5]. The spectrum of MD is well-summarized in a recent review [6].

In genotype-phenotype correlations in MDs, we should note two types of heterogeneities: (1) the same pattern of muscular dystrophies can be caused by mutations in different genes and (2) the different mutations in the same gene may cause different patterns of muscular dystrophy. In terms of pathogenic mechanism, myotonic dystrophy type 1 (DM1) and type 2 (DM2) and oculopharyngeal muscular dystrophy (OPMD) belong to a distinct group of muscular dystrophy caused by RNA gain-of-function from trinucleotide repeat expansion [2–4, 7–10], while facioscapulohumeral muscular dystrophy (FSHD) is caused by the contraction of microsatellite D4Z4 repeats [11], and the remaining MDs are caused by point mutations, deletions, duplications, and inversions [6]. Patents with MDs are often succumbed to a long arduous clinical course of progressive muscle weakness and wasting often resulting in significant disability and various complications. There is currently no cure for MDs, and available treatments are supportive care or of limited efficacy. Appropriate disease models are important for elucidation of disease mechanism and identification of treatment target.

## Models for the Study of Muscular Dystrophies Before the Emerging of iPSC Technology

Drosophila, zebrafish, and mammalian models (mouse, rat, hamster, and canine), including non-human primates, have all been adopted for the studies of muscular dystrophies. A large number of models have been developed and were reviewed elsewhere [6, 12, 13]. Taking DM1 as an example, approximately 20 mouse lines have been generated [9, 14, 15]. All these models have greatly enhanced our understanding of MDs and testing therapeutic approaches. However, they all have a common limitation; they are fundamentally non-human models with different genomic backgrounds. Furthermore, making animal models for each mutation that causes a particular MD for all MDs is not easily achievable due to time, effort, and cost. Human induced pluripotent stem cell (iPSC) model may fill these gaps. In this short review, we will summarize recent progress of using iPSCs as models for the studies of MDs.

## Human iPSC as Models for Disease Mechanism Studies and Drug Discovery of Muscular Dystrophies

The human iPSCs are generated by direct reprogramming of human somatic cells. These human iPSCs possess many of the properties of human embryonic stem cells (ESCs) and have the potential to differentiate into any type of cell or tissue in the body, including skeletal muscle cells and cardiomyocytes [16, 17•, 18–29]. The most anticipated clinical application of iPSC technology has been personalized cell therapy. While possible in principle, there are many hurdles to overcome (tumorigenicity, immunogenicity, immaturity, integration with existing cells in the tissue, and functional restoration) [29–31]. An immediate and practical application of iPSCs is to generate in vitro isogenic disease models. Disease-specific iPSCs will preserve the genetic mutations carried by the patient with the functional human genomic background, which cannot be accomplished in animal models. Disease-specific iPSCs can recapitulate disease features and potentially become a platform for drug development [32, 33]. Indeed, effective disease modeling with human iPSCs has been demonstrated in many inherited neurodegenerative disorders [32, 34–38], including MD (Table 1).

**Table 1:** MD iPSC lines discussed in this review

Muscular dystrophy	Study type	Published journal	First author (year)^ref^
DMD	Disease modeling	Cell. 134:877–886	Park et al. (2008) [39]
DMD	Therapeutic genome editing	Stem Cell Reports. 4:143–154	Li et al. (2015) [40]
DMD	Therapeutic genome editing	Mol Ther. 18:386–393	Kazuki et al. (2010) [41]
DMD	Disease modeling/cardiomyocytes	Int Heart J. 57:112–7	Hashimoto et al. (2016) [42]
DMD	Mechanistic study	Sci Rep. 5:12831	Shoji et al. (2015) [43]
DMD	Drug discovery	Stem Cells Transl. Med. 3:149–160	Abujarour et al. (2014) [44]
DMD	Mechanistic study/cardiomyopathy	Dis. Model. Mech. 2015	Lin et al. (2015) [45]
LGMD2B	Therapeutic genome editing	Mol Ther. 24:685–96	Turan et al. (2016) [46•]
LGMD2B	Disease modeling	PLoS One. (4):e61540	Tanaka (2013) [47]
FSHD	Mechanistic study	PLoS Genet. 6:e1001181	Snider et al. (2010) [48]
FSHD	Disease modeling	Stem Cells Transl Med. 5:1145–61	Caron (2016) [49]
LGMD2D	Cell-based therapy	Sci. Transl. Med. 2012;4	Tedesco et al. (2012) [50]
LGMD2D	Therapeutic genome editing	Mol Ther. 24:685–96	Turan et al. (2016) [46•]
LGMD2Z	Disease modeling	Stem Cell Research. 24:102–105	Wu (2017) [51]
LGMD2I	Mechanistic study/cardiomyopathy	Circ Genom Precis Med. 11:e001893.	El-Battrawy et al. (2018) [52]
DM1	Disease modeling	Cell Reprogram. 15:237–48	Xia (2013) [53]
DM1	Therapeutic genome editing	Stem Cells. 33:1829–38	Xia et al. (2015) [54]
DM1	Therapeutic genome editing	Mol Ther. 24:1378–87	Gao et al. (2016) [55]

Acquiring disease-specific iPSC is just the first step to model MDs. To successfully model the disease, iPSC will need to be differentiated into skeletal muscle progenitor cells (SMPCs) (satellite-like cells) and muscle fibers for study of muscle development and degeneration of a specific MD. The current limitation is to generate homogeneous SMPCs and to differentiate them into mature myofibers. Initially, the induction of skeletal muscle fibers from ESCs or iPSCs used spontaneous differentiation of embryoid bodies with conditional transgene overexpression of key myogenic factors (PAX7, PAX3, and MYOD1) [18, 22, 56, 57]. This strategy is not applicable for clinical application of cell-based therapy due to random integration of the exogenous DNAs, often using viral vectors, which raises an issue for potential insertional mutation [58]. More recently, serum-free and chemically defined induction by activation of Wnt signaling and/or inhibition of bone morphogenetic protein (BMP) signaling has been introduced and generated favorable results [16, 17, 20, 21, 59, 60]. In embryogenesis, Wnt-β-catenin activation specifies early paraxial mesoderm development, which subsequently gives to skeletal muscle, whereas BMP inhibition can prevent the newly specified paraxial mesoderm cells from drifting to a lateral plate mesoderm, which is a tissue that contributes to the long bones of the limbs but not skeletal muscle [61]. Wnt signaling drives the symmetric expansion of satellite stem cells [62]. Wnt-β-catenin signaling is negatively regulated by GSK3 [63]. GSK3 inhibitor (CHIR99021) is frequently used to activate Wnt signaling. With the understanding of above mechanism and the availability of cell signaling molecules, skeletal muscle differentiation protocols are becoming well established to acquire more homogeneous skeletal muscle progenitor cells and muscle fibers.

**Fig. 1:**
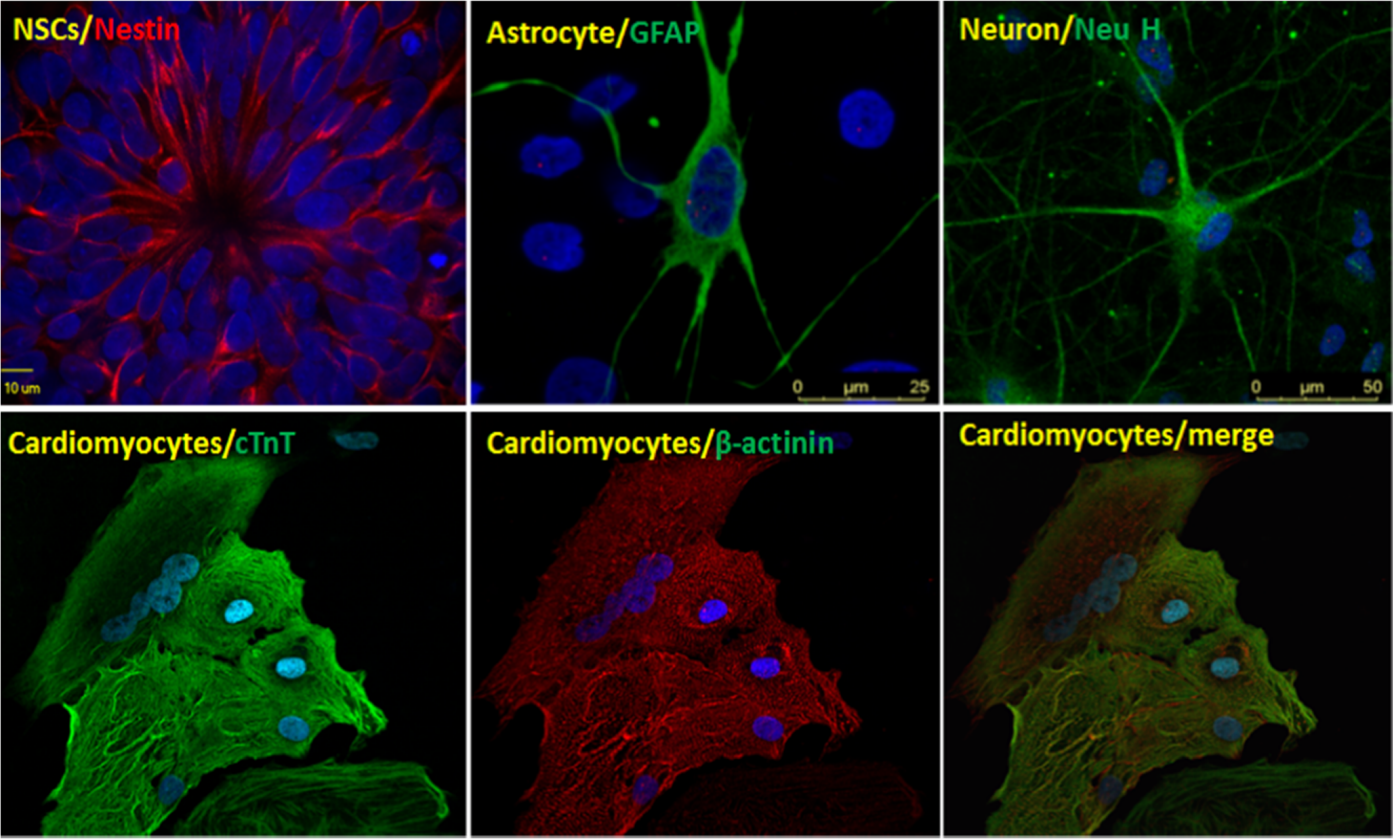
DM1 iPS cells derived neural stem cells, astrocytes, neurons, and cardiomyocytes

Even though skeletal muscle is the affected tissues for most muscular dystrophies, some MDs have multiple tissues and organs affected, for example, cardiomyopathy in DMD [64–66], EDMD [67, 68], and LGMD2I [69–71] and multisystemic involvement in DM1 and DM2 with progressive muscle wasting, myotonia, cardiac conduction defects, diabetes, gastrointestinal malfunction, and central nervous system impairment [4, 72–74]. The pluripotency of iPSC to differentiate to all somatic cell types makes it an attractive model. Neural and cardiac systems are tissues developed early in embryogenesis, and induced differentiation is relatively easy. The induction protocols are well-defined, and commercial kits are readily available. We routinely differentiate DM1 iPSCs into neural cells and cardiomyocytes, which show the typical hallmarks of intranuclear RNA foci in DM1 (Fig. 1). Other cells and tissue can also be generated from iPSCs to unveil the mechanism of the disease in different tissues.

Owing to this advancement, iPSC models have shed light on the pathogenesis of some MDs. DMD has been the focus of iPSC-based studies, from mechanistic studies and drug discovery to therapeutic genome editing and personalized cell-based therapy. The first DMD iPSC line was established in 2008 [39], which was generated from skin fibroblast carrying deletion of exon of 45–52 in the dystrophin gene. This iPSCs are confirmed to carry the disease-specific genotype of their parental cells. Since then, additional DMD iPSC lines have been established [40–42, 75]. The early pathogenic events in DMD can be effectively studied in skeletal myotubes induced from patient-derived iPSCs. In one study using iPSC-derived skeletal myotubes, the authors found control, and DMD myotubes derived from iPSCs were morphologically and physiologically comparable. However, electric stimulation of these myotubes caused pronounced calcium ion (Ca^2+^) influx only in DMD myocytes. Restoration of dystrophin by the exon-skipping technique suppressed this Ca^2+^ overflow and reduced the secretion of creatine kinase (CK) in DMD myotubes, suggesting the early pathogenesis of DMD can be effectively modeled in skeletal myotubes induced from patient-derived iPSCs [43]. Cardiac function is affected in all patients with DMD over 18 years of age and is becoming the most frequent cause of death [76]. The underlying mechanism of DMD-associated cardiomyopathy is not fully clarified due to the infeasibility to acquire live cardiomyocytes from the patients. Most of the studies were based on *mdx* mouse model. Various abnormalities have been reported in *mdx* mice [77–79]. However, *mdx* mice do not develop typical cardiac presentation in DMD patients [80, 81]. DMD patient-specific iPSCs can be successfully differentiated into contractile cardiomyocytes, which may recapitulate some of the human-specific abnormalities underlying the patient phenotype such as arrhythmias and conduction block [42]. In addition, further mechanistic studies could be attempted using the live DMD iPSC-derived cardiomyocytes for the understanding of DMD cardiomyopathy. Currently, there is no curative treatment for DMD cardiomyopathy. The unveiling of its pathogenesis will enable the development and evaluation of drug discovery.

LGMD2I is a dystroglycanopathy, caused by homozygous or compound heterozygous mutation in the *FKRP* gene (fukutin-related protein) [82]. Over 50% of patients had cardiac involvement (progressive dilated cardiomyopathy and ventricular tachycardia) [69–71]. The detailed molecular or electrophysiological mechanism is not defined because of the difficulties of accessing live human cardiac cells and animal models failed to demonstrate cardiomyopathy [83]. A recent study using human iPSC model shed light on the pathogenesis [52]. The author found that human iPSC-derived cardiomyocytes from a patient with LGMD2I (patient also has dilated cardiomyopathy associated with recurrent ventricular tachycardia) exhibited sodium, calcium, and K+ channel dysfunction, leading to reduced amplitude and upstroke velocity of action potentials as well as diminished Ca^2+^ release. The reduced upstroke velocity of action potentials may impair the conduction of the excitation in the heart and the rhythm. The diminished Ca^2+^ release may reduce contraction force of cardiomyocytes and cause dilated cardiomyopathy. This disease-specific human iPSC cardiomyocytes can thus provide a platform for studies on the cardiac events in LGMD2I and for drug discovery targeting cardiac myopathy.

The advantage of iPSC over primary culture of muscle cells is that it will mimic the developmental stage of muscle development and will help to understand whether the disease arises from developmental process or degenerative process. Facioscapulohumeral dystrophy (FSHD 1) is an autosomal dominant muscular dystrophy caused by the deletion of a subset of D4Z4 macrosatellite repeat units in the subtelomeric region of 4q on the 4A161 haplotype (FSHD 1). FSHD 1 iPSC lines were established for the disease mechanism studies [48]. Using the disease-specific human iPSCs, the key function and implication of mRNA and protein of *DUX4* in FSHD 1 were able to be studied from the early development. The author was able to confirm their findings in other model system of the developmental regulation of DUX4 and their role in FSHD. They found that the transition between DUX4 full-length and DUX4 short-length expression is developmentally regulated. DUX4 short-length, but not DUX4 full-length, was detected in control fibroblasts. iPSCs derived from the control fibroblasts expressed DUX4 full-length, whereas differentiation of these cells to embryoid bodies resulted in a switch to the expression of DUX4 short-length and loss of DUX4 full-length. In contrast, DUX4 full-length was detected in FSHD fibroblasts and the iPSCs and embryoid bodies derived from FSHD fibroblasts. DUX4 full-length was detected in some human ES cell lines, but at much lower levels compared to the iPSCs. They concluded that full-length DUX4 mRNA is normally expressed early in development and is suppressed during cellular differentiation, whereas FSHD is associated with the failure to maintain complete suppression of full-length DUX4 expression in differentiated skeletal muscle cells.

The disease mechanism in skeletal muscle in MDs has been well-studied by mouse models and fibroblast/myoblast cell cultures (see reviews) [2, 4, 7–9]. However, some MDs have multiple systems affected as mentioned above. For example, the CTG repeat expansion in DM1 also caused symptoms in the central nervous system (CNS) and the mechanism is less defined. A major obstacle is difficulty in obtaining viable tissues in the CNS. The clinical studies have been largely restricted to investigations of clinical findings, neuropsychiatry, neuroradiology, and neuropathology [84–98]. And the underlying molecular mechanism for CNS involvement in DM1 has been explained as a “*spliceopathy*.” Abnormal splicing of microtubule-associated protein tau (*MAPT*) gene has been identified in DM1 brain with corresponding pathological findings of neurofibrillary tangles [98–104]. Studies of transgenic and knockout mouse models suggested that sequestration and loss of function of *mbnl 2* appears to play a major pathogenic role in the DM1 brain pathology [105]. To further advance these studies, iPSCs can provide an unlimited resource suitable for electrophysiological and interventional mechanistic experiments in the human genomic environment in many different cell types, including neuronal and glial lineages. We have generated disease-specific DM1 iPSC lines. These cells harbor the naturally mutated gene in the same genomic background. We have been able to differentiate these iPSC lines into neural stem cells, neurons, astrocytes, and cardiomyocytes and skeletal muscle fibers, which all showed intranuclear RNA foci and aberrant splicing, faithfully representing DM1 phenotypes [54, 55]. We think this is an isogenic cellular model for mechanistic study for this multisystemic disease and for therapeutic drug discovery.

Drug screening for MDs was traditionally conducted in primary culture of myocytes or immortalized myoblast cells. iPSC as models for high-throughput drug screening has been conducted in many other diseases. Methods for the differentiation of iPSCs into skeletal muscle fibers and cardiomyocytes have been developed as reviewed above. The advantage over other cell types is to generate consistent cell population unlimitedly. The results are more translatable to clinical application. DMD iPSC is a good example for drug discovery. iPSC from DMD patients have been differentiated into dystrophic myotubes and cardiomyocytes, and therapeutic drug has been tested [44, 45]. Methodology has also been developed for high-throughput drug screening [106].

One other advantage of iPSC is to establish isogenic cellular model by incorporating genome editing to correct the mutation. With the development of deep sequencing, RNA sequencing, and bioinformatics, more valuable information can be extracted from the pairwise comparison of the big sequencing data among normal iPSC, disease-specific iPSC, and genome-corrected iPSC-derived specific cell types. We have also endeavored to create this isogenic cellular model for DM1 [107].

Moreover, toxicity can also be evaluated in these isogenic DM1 cellular models. Cardiotoxicity and neurotoxicity are the main reasons for some drugs to fail clinical trials. These are traditionally tested in animal models due to the hard-to-get live human cardiomyocytes or neurons. This is changing with the advancement of iPSC technology and may affect regulation for drugs to get into clinical trials. The efficacy on iPSC-derived specific cell types and toxicities may be listed as a key step before moving a therapeutic drug to clinical human trials.

New iPSC models for MD are quickly emerging. Recently, an iPSC line from a new type of LGMD (LGMD2Z, OMIM#617232), which is caused by a missense mutation in *POGLUT1* [108], has just been established [46•]. We are expecting more muscular dystrophies will be developed to serve as in vitro disease models for identification of pathogenesis and therapeutic targets.

## iPSC as Models for Development of Personalized Cell-Based Therapy for MDs

Skeletal muscle cell transplantation for muscular dystrophy was previously tested on DMD. However, the results were disappointing. The main issue was the source of the transplanted cells. All early studies used allogenic myoblasts derived from muscle biopsy tissues. The initial immune reaction killed 75–80% of the transplanted cells [109–115]. Besides, myoblasts have their own intrinsic defects for cell-based therapy. Myoblasts are acquired from in vitro culture of isolated satellite cells from the muscle tissues. These myoblasts can only proliferate for a limited number of passages, and further ex vivo expansion degrades their myogenic capacity [116]. Upon transplantation, survived myoblasts migrated poorly and failed in replenishing the satellite compartment and the effect cannot be sustained [116, 117]. Other human muscle stem cells have been investigated for cell-based therapy [118–124], but they need to be isolated from live human muscle tissues. Large quantities of cells are needed for autologous cell transplantation therapy. Unfortunately, to manufacture a therapeutic quantity of muscle stem cells from a MD patient’s muscle tissue is almost impossible without causing severe, permanent damage to the already-atrophied muscle.

With the emergence of iPSC technology, the above issues are being resolved [24, 25, 125]. There has been increasing enthusiasm about applying iPSC technology to generate autologous cells for therapeutic purposes [126–133], and the first human trial for macular degeneration has been conducted with encouraging results [134]. The advantage of iPSC is the prospect of generating unlimited quantities of specific cell population for regenerative purposes. iPSCs are derived from somatic cells and do not involve the use of embryo, and there is no ethical concerns. iPSCs generated from the same patient, termed patient-specific iPSCs, can theoretically avoid immune rejection [24, 25, 125]. Cell transplantation has been conducted in mouse models of DMD and LGMDs. These cells are able to fuse to host myofibers and exhibit good strength. These cells were also able to seed the muscle satellite cell compartment [20, 50, 56]. This is of in particular importance as continuous cycles of myofiber degeneration and regeneration in advanced degenerative muscular dystrophy may exhaust the satellite cell reserves and thus lose their regenerative capacity [135–137]. Restoration of the satellite cell pool will restore the regenerative capacity of the muscle and maintains sustained effects.

However, patient-derived iPSCs still carry the mutation that is causative for MDs, and myogenic cells derived from these iPSCs may undergo the same degenerative process after transplantation. To overcome this, approaches have been developed to correct the mutation to restore the expression of lost proteins for the purpose of cell transplantation [40, 41, 50, 138].

### Genome Correction for Autosomal Recessive Point Mutation Genes

Limb-girdle muscular dystrophy 2D (LGMD2D) is caused by mutations in the gene encoding α-sarcoglycan. Four iPSC lines have been established form patient fibroblasts and myoblasts [50]. The authors differentiated iPSCs into mesoangioblasts-like mesodermal progenitor cells, which can be further differentiated into muscle fibers. To genetically correct LGMD2D iPSC-derived mesoangioblasts, the authors developed a new lentiviral vector carrying the human α-sarcoglycan cDNA under transcriptional control of the muscle-specific myosin light chain 1F promoter and enhancer. The transgene is selectively expressed in myotubes generated from genetically corrected LGMD2D mesoangioblasts. They showed that it is possible to reprogram adult somatic cells from LGMD2D patients to pluripotency and to genetically correct mesoangioblasts derived from LGMD2D iPSCs. They also showed that the genetically corrected mesoangioblasts derived from LGMD2D iPSCs undergo terminal myogenic differentiation with correct and specific expression of the therapeutic transgene. When these genetically corrected human iPSC-derived mesoangioblasts were transplanted into α-sarcoglycan-null immunodeficient mice, they generated muscle fibers that expressed α-sarcoglycan. Finally, transplantation of mouse iPSC-derived mesoangioblasts into α-sarcoglycan-null immunodeficient mice resulted in functional amelioration of the dystrophic phenotype and restoration of the depleted progenitors. This is not a true therapeutic genome editing. The original mutation in the genome remains unchanged. The current technology now allows us to correct the mutation in situ in the mutated gene (*SGCA* in LGMD2D) [46•]. This disease-specific iPSC model will be ideal to test the correction strategies.

In a recent publication, a research group from Stanford University reported strategies to correct the mutation in MD iPSC lines [46•]. They successfully corrected dysferin nonsense mutation in LGMD2B c.5713C>T; p.R1905X and the most common alpha-sarcoglycan mutation in LGMD2D, missense c.229C>T; and p.R77C, by homology-directed repair enhanced by a site-specific double strand break using CRISPR/Cas9 gene-editing system. For each mutation in the same gene that caused the loss of gene function, a specific correction needs to be investigated and validated, which decreases its feasibility in clinical application. As an alternative approach for these MDs mediated by protein loss-of-function, the authors suggested insertion of wild-type gene into the H11 safe harbor or AAVS1 site using dual integrase-assisted exchange (DICE) or TALEN/CRISPR/Cas9-assisted homologous recombination may offer a more versatile approach.

### Genome Correction for Autosomal Dominant Muscular Dystrophy

We also explored strategies to correct the mutation in an autosomal dominant MDs, DM1, a disease of RNA gain-of-function. In DM1, the abnormal myogenesis of myoblasts from DM1 patients [139–147] prevents them from being used as an ideal source for cell transplantation therapy. To circumvent this hurdle, we have succeeded in editing the genome to eliminate the expanded CUG mutant transcripts via precise incorporation of polyadenylation signal upstream of the *DMPK* CTG repeats. The polyadenylation signals prematurely terminate the transcription upstream of the expanded CTG repeats. Genome-edited human DM1 iPSCs maintain their pluripotency, and their neural and cardiomyocyte derivatives all lost nuclear RNA foci and demonstrated reversal of aberrant splicing [54, 55]. We have further improved the strategy by insertion of polyadenylation signals in the 3′-UTR between the stop codon and expanded CTG repeats, which generated full-length *DMPK* protein. These genome-edited human DM1 iPSCs can be differentiated into skeletal muscle progenitor cells (SMPCs). We hypothesize that these SMPCs can engraft and repopulate the muscle tissue to restore muscle function. Other groups have tried to delete the disease-causing CTG repeats by dual sgRNA/CRISPR-Cas9 flanking the CTG repeats [148, 149]. However, we found frequent inversion of the flanked CTG repeats (our unpublished data). This approach may be used to establish isogenic cell model by selecting clones which have pure deletion, but will not be a viable for in vivo therapeutic therapy.

### Genome Correction for X-Linked DMD/BMD

Approaches to restore dystrophin expression in DMD iPSC lines have been established [40, 150]. Dr. Akitsu Hotta’s and Shinya Yamanaka’s group in the Center for iPS Cell Research and Application did the first pioneering work published in 2014. They tested exon skipping, frameshifting, and exon knock-in in DMD-patient-derived iPSCs using TALEN and CRISPR technologies and found that exon 44 knock-in was the most effective approach. The corrected iPSCs were differentiated toward skeletal muscle cells and successfully detected the expression of full-length dystrophin protein [40]. DMD has a wide range of over thousand mutations, and designing individual correction method seems impractical. Recently, a group in UT Southwestern used CRISPR/Cas9 with single-guide RNAs to destroy the conserved splice acceptor or donor sites preceding DMD mutations or to bypass mutant or out-of-frame exons, thereby allowing splicing between surrounding exons to recreate in-frame dystrophin proteins lacking the mutations and was able to rescue dystrophin function in up to 60% of DMD patients [151]. In this study, they also tested the efficacy on engineered heart tissue from human iPSCs. They were able to demonstrate that correcting only a subset of cardiomyocytes (30 to 50%) was sufficient to rescue the mutant phenotypes to near-normal control levels.

## Challenging Issues of iPSC Models

Our musculature is composed of many types of muscle in the body: cranial muscle, trunk muscle, and limb muscle. They have different developmental origins and programs. Each muscle is composed of slow or fast myofibers expressing different types of myosin heavy chain genes. To faithfully mirror the physiology and pathology in vivo, such differences should be considered. However, an induction method for diverse types of myofibers is at present challenging. Maturation of skeletal muscle fibers derived from human iPSCs using current in vitro protocols is generally limited. We have tried multiple published differentiation protocols, including direct induction by transfection of key myogenic factors and chemically induced protocols. We were able to get MHC-expressing myofibers, but we have not been able to generate mature multinucleated myofibers as we can normally see with myoblasts isolated from muscle biopsies (unpublished data). This is partly due to lack of innervation to the myofibers. This is an issue to model disease, but these nascent myofibers could be a good cell source for cell transplantation therapy. As disease models, the most significant limitation is that iPSC offers a cellular models but not in vivo models. The development of organoids using iPSC technology will allow us to study the disease in tissue or organ level, but they are still not recapitulating the entire organism. In terms of the use of iPSC in cell transfer therapy, challenge issues include delivery of iPSC-derived genome-edited cells to a large mass of muscles, GMP production of a therapeutic amount of these cells, immunological reactions for transferring cells expressing the deficient protein in loss-of-function MDs, and the frequency of cell transfer therapy to replenish therapeutic cellular populations.

## Conclusions

In this short review, we first introduced the background of MDs and iPSC. We reviewed briefly the methodology of myogenic differentiation from iPSCs. We then reviewed the application of human disease-specific iPSC models in mechanistic studies, drug screening, and personalized cell-based therapy. In summary, human disease-specific iPSC models for MDs are great addition to our current armamentarium for elucidation of disease mechanism and therapeutic development.
